# Achieving Gameplay Independence in Virtual Reality Exergames for Individuals With Mild Intellectual Disabilities: Pilot Study

**DOI:** 10.2196/71823

**Published:** 2025-11-14

**Authors:** Julia Ciążyńska, Janusz Maciaszek

**Affiliations:** 1 Department of Physical Activity and Health Promotion Science Akademia Wychowania Fizycznego im. Eugeniusza Piaseckiego w Poznaniu Poznan, Greater Poland Voivodeship Poland

**Keywords:** active video game, exergame, gameplay experience, health promotion, independence, intellectual disabilities, physical activity, physical education, usability, virtual reality

## Abstract

**Background:**

Individuals with mild intellectual disabilities (ID) often face cognitive and functional challenges, which can lead to low physical activity (PA) and a higher risk of obesity. While virtual reality (VR) exergames show promise for promoting PA in typically developing children, a key barrier for individuals with ID is the lack of a structured teaching methodology. This study argues that a tailored approach is essential to help children with mild ID gain independence in gameplay. By learning specific patterns, they can achieve greater autonomy, which not only facilitates increased PA but also improves motor competence, physical fitness, functional abilities, and overall well-being.

**Objective:**

This study aims to evaluate the effectiveness of the WISH (Warm-up, Imitation, Settings, Half-hour exergame session) and WON (Warm-up, Objective evaluation, No problem!) training protocols in improving participant independence, exergame performance, and overall gameplay experience in VR.

**Methods:**

We used a multisession, single-group research design involving 16 training sessions in this pilot study. The 16 sessions were conducted during scheduled physical education classes at a special school in Poland from October 2023 to May 2024. The intervention comprised two main protocols: the WISH protocol (sessions 1-4), an introductory phase focused on familiarization with VR technology and gameplay mechanics, and the WON protocol (sessions 5-16), designed for gradual reduction of trainer assistance to promote gameplay independence.

**Results:**

The statistical analysis confirmed the effectiveness of both the WISH and WON protocols. A Wilcoxon signed-rank test on the WISH protocol revealed a statistically significant improvement in understanding instructions for the warm-up (*r*=0.87; *P*=.009), the projector imitation (*r*=0.91; *P*=.007), and participant exergame performance (*r*=0.90; *P*=.03). Within the WON protocol, the Wilcoxon test also showed a significant increase in participant exergame performance (*r*=0.89; *P*=.008). Further analysis using Spearman rank-order correlation indicated a very strong association between increased independence and better exergame performance (ρ=0.91; *P*=.002) and overall gameplay experience (ρ=0.63; *P*<.05).

**Conclusions:**

This pilot study suggests that the structured WISH and WON training protocols may have the potential to enhance functional autonomy, exergame performance, and overall gameplay experience in individuals with mild ID. The observed improvements indicate that such structured pedagogical approaches could be beneficial for this population. These preliminary findings warrant further investigation through larger-scale, controlled studies to confirm efficacy and explore the transferability of these benefits to broader contexts and other VR exergames.

## Introduction

Intellectual disabilities (ID) comprise a spectrum of severity levels: mild (F70), moderate (F71), severe (F72), and profound disability (F73), as per the International Classification of Diseases, 10th Revision, by the World Health Organization [1]. Mild severity is characterized by an intelligence quotient (IQ) between 50 and 70, moderate by an IQ between 35 and 49, severe by an IQ between 20 and 34, and profound by an IQ of less than 20 [1]. Individuals with mild ID have difficulties in many cognitive and functional abilities [2]. They may struggle with expressing their thoughts, retaining information, interpreting sensations [3], and engaging in both fine and gross motor movements and motor competence [4]. Despite these obstacles, most people with mild ID can lead productive lives with some assistance. This category accounts for about 85% of those with ID and may need help with some areas of life but remain at least partly independent [5]. People with mild ID tend to be less physically active than their peers without disabilities [6]. This reduced physical activity (PA) can contribute to poor physical health, including low cardiovascular fitness, weak muscles, and an increased risk of overweight and obesity. Given these challenges, developing effective strategies to increase PA participation among individuals with ID is crucial [7]. By promoting regular exercise, we can help improve their overall health and well-being.

Recent initiatives to promote PA among typically developing children have involved incorporating exergames into school physical education (PE) classes [8-10]. These exergames use innovative technology to create immersive environments that require physical gestures and movements to control the gameplay. Additionally, fully immersive virtual reality (VR) exergames transport users into the game world, often through head-mounted displays, eliminating distractions and focusing attention on the game. Exergames incorporating interactive technology and immersive VR experiences have shown promise in promoting PA for people with ID [11-13]. However, before the potential gains of PA exergames that people with ID may derive from exergaming are examined, a teaching methodology should be developed that will combine manual and digital techniques for teaching correct movement patterns [14,15]. Knowing these patterns and practicing them can help improve general physical fitness and functional ability and prevent injury [14]. Consequently, an appropriate step-by-step guide [16] should be developed for introducing people with ID to the VR exergame to achieve independence in gameplay [17]. Recent research suggests that technology systems designed to provide step-by-step guide instructions can be effective tools for teaching and supporting individuals with ID to independently complete tasks and develop motor competence [17]. Building independence in the sphere of exergaming with occasional help from a PE teacher can translate into a sense of independence in taking actions in everyday life for people with ID. Independence, like self-reliance, is a term that involves the ability to take actions to manage one’s affairs and provide for oneself [18]. This entails relying on one’s own efforts, resources, judgment, and abilities without requiring support from others. PE teachers can aim to guide people with ID toward the most optimal level of independence that is attainable for an individual.

Beyond the specific context of ID, the creation of standardized methods for teaching VR goggle use is a critical and unmet need in the broader fields of PE and rehabilitation. While the potential of VR to enhance engagement in PA and therapeutic exercises is widely recognized [19], its effective application is often limited by the lack of structured instructional protocols. Other studies, such as that by Villada Castillo et al [20], have outlined protocols for introducing exergames for stroke rehabilitation, focusing on a brief introduction, system setup, and free play to gather feedback. Similarly, a methodology proposed by Pirovano et al [21] emphasizes a 3-step design process for therapeutic exergames: identifying exercise requirements, transforming exercises into a virtual environment, and adding game elements for entertainment.

This study builds upon this work by providing a comprehensive teaching methodology that is uniquely structured to facilitate independence. Unlike existing protocols that are often brief or focused on system design and feedback gathering, our approach integrates with a standard PE lesson structure. This division into a warm-up, main part, and cool-down over 45 minutes addresses the practical needs of a school setting, ensuring that the protocols are not only effective but also highly adaptable. By providing a successful protocol for teaching the use of VR technology, our research aims to address this gap. Creating tailored, step-by-step methods is essential for making VR an accessible and effective tool, ultimately fostering greater engagement in activities crucial for well-being across a wide spectrum of users.

Despite evidence that VR exergaming can increase PA, to the best of the authors’ knowledge, no previous research has reported on a methodical training protocol for introducing VR gameplay into PE lessons, specifically designed for individuals with mild ID.

The aim of this study was to evaluate the effectiveness of the WISH (Warm-up, Imitation, Settings, Half-hour exergame session) and WON (Warm-up, Objective evaluation, No problem!) training protocols in improving participants’ independence, exergame performance, and overall experience playing a VR exergame.

This pilot study aimed to explore whether (1) a structured teaching methodology could be beneficial for implementing VR technology in individuals with mild ID, (2) the proposed protocols could lead to improvements in participants’ functional independence and sense of motor competence, and (3) the proposed protocols show potential to serve as a transferable approach for teaching other VR games based on similar movement mechanisms, warranting further investigation.

## Methods

### Ethical Considerations

The institutional review board at the Karol Marcinkowski Medical University in Poznań approved this study (no 684/23). All procedures were conducted in accordance with the ethical standards of the institutional and national research committees and with the 1964 Helsinki Declaration, and its later amendments or comparable ethical standards.

All participants were pupils in the special school complex in Poland. Prior to their involvement, comprehensive written informed consent was obtained from the parents or legal guardians of all participants. Additionally, each participant provided verbal and written assent, ensuring their voluntary agreement to take part in the study. Participants did not receive any monetary compensation for their involvement.

To ensure the privacy and confidentiality of participants, all collected data were anonymized and deidentified immediately upon collection. Unique numerical codes were assigned to each participant, and all personally identifying information was stored separately in a secure, password-protected database accessible only to the principal investigator. No personally identifiable data were included in the research database or in any reports or publications.

Individuals visible in the table-of-contents picture are either the authors or pupils who participated in the study. Explicit written consent for the publication of their identifiable images was obtained from these individuals or their legal guardians. These consent forms have been uploaded as supplementary material alongside the manuscript for verification.

### Study Design

The WISH protocol (Textbox 1) and the WON protocol (Textbox 2) were developed as a coherent training methodology to gradually increase independence in children with mild ID when using VR exergames. Their names are acronyms that reflect the structure and purpose of each stage. The WISH and WON protocols were initially tested on a sample of healthy adults to identify foundational inconsistencies and refine the core procedures. Subsequently, the protocols were evaluated in a cohort of healthy children aged 15 years to detect and address specific challenges related to a younger population, ensuring the methodology was appropriately adapted for the target group. Finally, before being used, the protocols were evaluated by a school psychologist, a PE teacher from a special school, and an impartial researcher, who gave positive opinions based on their knowledge and the potential usefulness of the protocols.

The WISH (Warm-up, Imitation, Settings, Half-hour exergame session) protocol is a 4-session introductory step. It was created to familiarize participants with the basics of VR exergames, acclimating them to the technology and gameplay mechanics with support. The WON (Warm-up, Objective evaluation, No problem!) protocol is the main one, running from session 5 to 16. It focuses on gradually reducing trainer assistance to promote full independence in gameplay. The name “No problem!” refers to the questionnaire used to measure the level of independence.

Both protocols were developed based on existing physical therapy and special education methodologies, adapted for the specifics of VR, and were validated through the continuous evaluation of participant progress across successive sessions.

**Figure 1:**
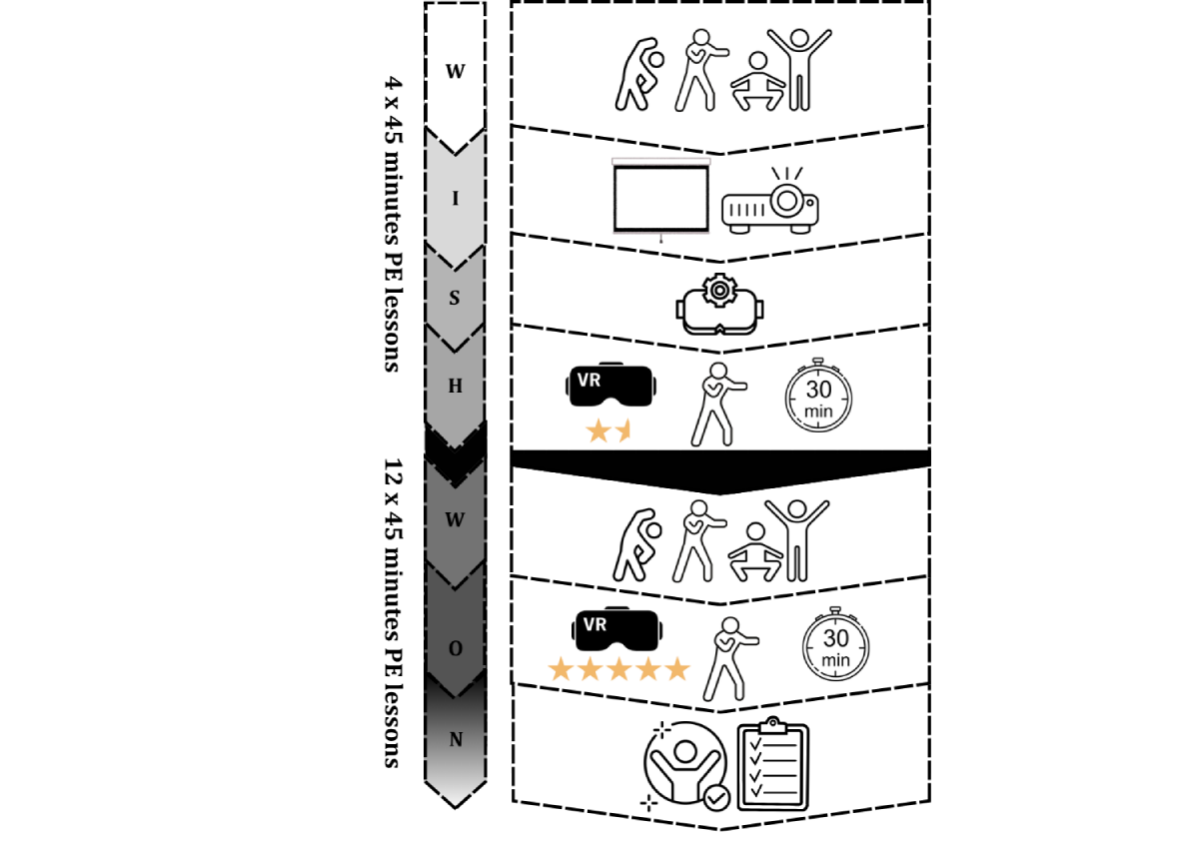
Overall design of the WISH (Warm-up, Imitation, Settings, Half-hour exergame session) and WON (Warm-up, Objective evaluation, No problem!) structured teaching protocols, illustrating their sequential intervention delivery over 16 biweekly sessions in a pilot study involving 8 adolescents with mild intellectual disabilities. The intervention was conducted at a special school in Poland from October 2023 to May 2024, spanning an 8-month period within the 2023-2024 academic year. PE: physical education; VR: virtual reality.

Each of the 16 WISH and WON sessions was conducted once every 2 weeks during PE classes at a special school, following a standard lesson structure of a warm-up, a main part, and a cool-down. The duration of a single exergame session (the exergame “OhShape!” was used) was approximately 30 minutes, with each protocol having a defined time structure for its respective stages. Overall graphical information for both protocols is available in Figure 1.

In OhShape!, users engage in a rhythmic fitness game with musical elements, in which the goal is to match their bodies to the shapes that appear on walls. Players stand before colorful walls with silhouettes cut into them, and their task is to position themselves correctly to pass through. The game also features additional elements, such as special walls that must be avoided by squatting or broken with the hands, and coins that are collected by performing movement sequences. The game engages the entire body, requiring players to perform squats, dodges, and dynamic poses to the rhythm of the music. This effectively combines fun with PA, offering a compelling fitness experience. A video of the “I” step with OhShape! exergame gameplay is provided in Multimedia Appendix 1.

**WISH (Warm-up, Imitation, Settings, Half-hour exergame session): a 4-step protocol (sessions 1-4).**

**Step 1. “W” (10 minutes)**

- The warm-up included movements inspired directly by in-game elements, designed to prepare participants for the main part of the training. The exercises performed were:
  arm swings (in-game: catching coins)
  hand movements imitating boxing punches (in-game: smashing walls)
  squats (in-game: avoiding walls that appear above the head)
  step-and-reach (in-game: avoiding walls)
  a “mirror” game, consisting of imitating movements performed by the trainer

**Step 2. “I” (3 minutes)**

- Imitating the exergame by using a projector and video recorded from the perspective of a participant in virtual reality (VR) goggles:
  the movements learned during the warm-up are used
  all participants position themselves in front of the image displayed by the projector and “play” without using VR goggles
  the trainer verifies the physical abilities of the participants and their understanding of the instructions. He reminds them aloud what to do with the appropriate element of the exergame (eg, avoid the transparent wall—do a squat; break the red wall—use boxing with your hands).

**Step 3. “S” (2 minutes)**

- Setting up the VR goggles and the exergame zone:
  marking the goggles with the participant symbol (A-H) and adjusting the goggles to the participants (setting the distance between the lenses, fastening straps)
  setting a safe zone for the participant in which they could move freely
  connecting the view from the goggles to the app on the trainer’s phone, which allowed for a real-time view of the exergame

**Step 4. “H” (30 minutes)**

- Half-hour exergame session:
  launching the game remotely for participants (the “A” button on the controller allowed voice launch of game elements through a voice message, eg, “launch game,” “again,” and “menu”)
  the game was launched at the lowest level of difficulty (“try out”)
  game control by viewing the progress of participants in the gym and from their perspective in the goggles through an app on the phone
  overall scores were recorded by the trainer (stars)

**WON (Warm-up, Objective evaluation, No problem!): a 3-step protocol (sessions 5-16).**

**Step 1. “W” (10 minutes)**

- The warm-up included more advanced movements directly inspired by in-game elements, designed to prepare participants for the main part of the training. The exercises performed were:
  arm swings (in-game: catching coins)
  both hands movements imitating boxing punches (in-game: smashing walls)
  squats (in-game: avoiding walls that appear above the head)
  step-and-reach (in-game: avoiding walls)
  combo movement combining squats and step-and-reach exercises
  a “mirror” game, consisting of imitating movements performed by the trainer

**Step 2. “O” (30 minutes)**

- Objective evaluation of exergame performance:
  based on the overall score (stars) obtained in the earlier session, the level of difficulty of the game on the day of the session was selected for each participant separately
  participants were not helped in the game unless they asked for it
  the exergame was not launched for the participant, they launched it themselves

**Step 3. “N” (5 minutes)**

- No problem! questionnaire:
  each participant completed the questionnaire and interview sessions independently

### Study Sample

All participants (N=8) were approached on the basis that they were pupils of a special school and had a mild intellectual disability (based on data provided by the diagnosis carried out in psychological and pedagogical counselling centers, aimed at recognizing the disability, determining the degree of ID, and issuing a decision on the need for special education). This was a convenience sample, comprising all eligible students from 2 distinct classes at the specific special school who provided parental/guardian informed consent and their own assent to participate. These 2 classes (totaling 8 pupils) represented the entire available cohort of students within the specified age range and disability level who typically participate together in PE lessons at this institution. Due to the pilot nature of the study and the specificity of the population, formal sample size calculations were not performed. The PE teacher and psychologist of their school assessed the level of physical fitness and mental health as good, enabling participation in the study. All participants exercise regularly in PE lessons in their special school.

A total of 8 participants (male: n=3; female: n=5) aged between 15 and 20 years (mean age 17.63 years, SD 1.77) took part in the study.

Inclusion criteria were as follows: no previous VR experience, mild ID, current pupils of a special school, teenagers or young adults aged 15-25 years, wearing or not wearing corrective glasses (a special overlay for glasses was applied), right- or left-handed, no use of psychotropic medicines, absence of long-term or recent injuries, and good physical fitness and mental health to participate in PE lessons (confirmed by a statement from a school psychologist and the current PE teacher of the special school).

Exclusion criteria were as follows: previous VR experience, no mild ID, former pupils of a special school, age greater than 25 years, history of eye surgery, use of psychotropic medicines, long-term or recent injuries, and below-average physical fitness or mental health to participate in PE lessons (as confirmed by a statement from a psychologist and the current PE teacher of the special school).

### Measures

#### Technology

A VR Meta Quest 2 128 GB system (Meta Platforms Technologies Ireland Limited, 2023) was used as the VR headset in this study. This system includes a wireless headset for viewing and exergaming, as well as 2 hand controllers. To ensure optimal performance, a minimum room size of 2.0 × 2.0 meters is recommended. The VR headset features a 5.7-inch OLED display with a resolution of 1832 × 1920 pixels per eye and a refresh rate of 60-90 Hz. The exergame used in this study, OhShape!, was downloaded from the Meta Quest Store app.

#### Procedure

The study was conducted over 16 sessions held as part of PE lessons at a special school. The entire procedure was based on two protocols, WISH and WON, which were designed to gradually increase participants’ independence in using a VR exergame.

The WISH protocol comprised 4 steps aimed at developing correct movement technique and ensuring safety and comprehension of the game’s rules. In the initial “W” (Warm-up) and “I” (Imitation), the trainer assessed whether participants understood the instructions and performed the movements correctly, recording the results on a task sheet (task sheet with 3-point scale: “YES,” 2 points, indicated complete understanding and correct execution; “PARTIALLY,” 1 point, indicated partial understanding or execution requiring minor cues; and “NO,” 0 points, indicated no understanding or incorrect execution). These scores were based on predefined, detailed criteria, ensuring consistent assessment across sessions and participants. During the “S” (Settings), the trainer ensured participant safety by setting up the VR goggles and play zones, documenting the settings to maintain consistency between sessions. The final stage, “H” (Half-hour exergame session), involved a 30-minute exergame gameplay segment during which the trainer remotely launched the game and monitored participant progress, recording their score (stars; exergame performance). The WISH protocol lasted 4 sessions, with the objective for each participant to achieve full understanding of the instructions (“W” and “I” stages) and earn at least 1.5 stars in the game.

The WON protocol was implemented following the 4 WISH sessions, with the primary objective of developing full independence in gameplay. The first step, “W” (Warm-up), continued to support correct movement technique. In the subsequent “O” (Objective evaluation) stage, participants transitioned to independent play, where they were responsible for launching the exergame themselves. The trainer adjusted the difficulty level individually for each participant based on their exergame performance (stars) from previous sessions and provided assistance only when requested. Participant progress or regression was monitored by recording their overall exergame performance score on a task sheet. The final step, “N” (No problem! questionnaires), involved a mixed methods assessment using the No problem! independence questionnaire, which contained 7 functional levels, and a separate gameplay experience questionnaire with a 3-point Likert scale.

### Independence and Gameplay Experience Questionnaires

The independence questionnaire, titled No problem!, was combined with interviews and used an adapted 7-level functional independence scale, directly derived from the FIM levels of the Functional Independence Measure (FIM) [22,23]. This adapted scale ranged from total assistance to total independence. Data on independence level were collected from training sessions 5 to 16. For clarity and transparency, the specific definitions of these adapted 7 functional levels are provided in Multimedia Appendix 2.

In this study, the definition of the 7 functional levels and corresponding 7-point scale was described as follows. A score of 7 points indicated total independence, where all tasks were completed without the need for assistance from third parties. In the interview, participants asked about problems during the exergame played used the phrase, “I had no problem.”

A score of 6 points reflected incomplete independence. The activity required longer (than expected) time to complete the gameplay, but the rest of the gameplay was correctly followed. In the interview, participants asked about problems during the exergame played used, for example, the phrase, “I had some problems, but I didn’t need supervision.”

A score of 5 points referred to supervision. The participant required no more assistance than standing next to a third person, including assignment, guidance, or persuasion to perform the activity, but no physical contact. In the interview, participants asked about problems during the exergame played used, for example, the phrase, “I had a few problems, and I needed a voice support.”

A score of 4 points indicated minimal assistance. Regarding physical contact, the participants required no more assistance than touching and performing most (over 75%) of the activity. In the interview, participants asked about problems during the exergame played used, for example, the phrase, “I had a few problems, and I needed minimal physical support.”

A score of 3 points represented moderate assistance, where the participant required more assistance or performed half or more of the activity/effort (50%-74%). In the interview, participants asked about problems during the exergame played used, for example, the phrase, “I had a few problems, and I needed moderate physical support.”

A score of 2 points reflected maximum assistance. The participant performed half or less of the specified activity (25%-49%), but at least 25%. In the interview, participants asked about problems during the exergame played used, for example, the phrase, “I had a lot of problems, and I needed a moderate physical support.”

A score of 1 point indicated total assistance. The participant could perform little, if any, of the planned activity/effort (less than 25%). In the interview, participants asked about problems during the exergame played used, for example, the phrase, “I had a lot of problems, and I needed total assistance.”

**Figure 2:**
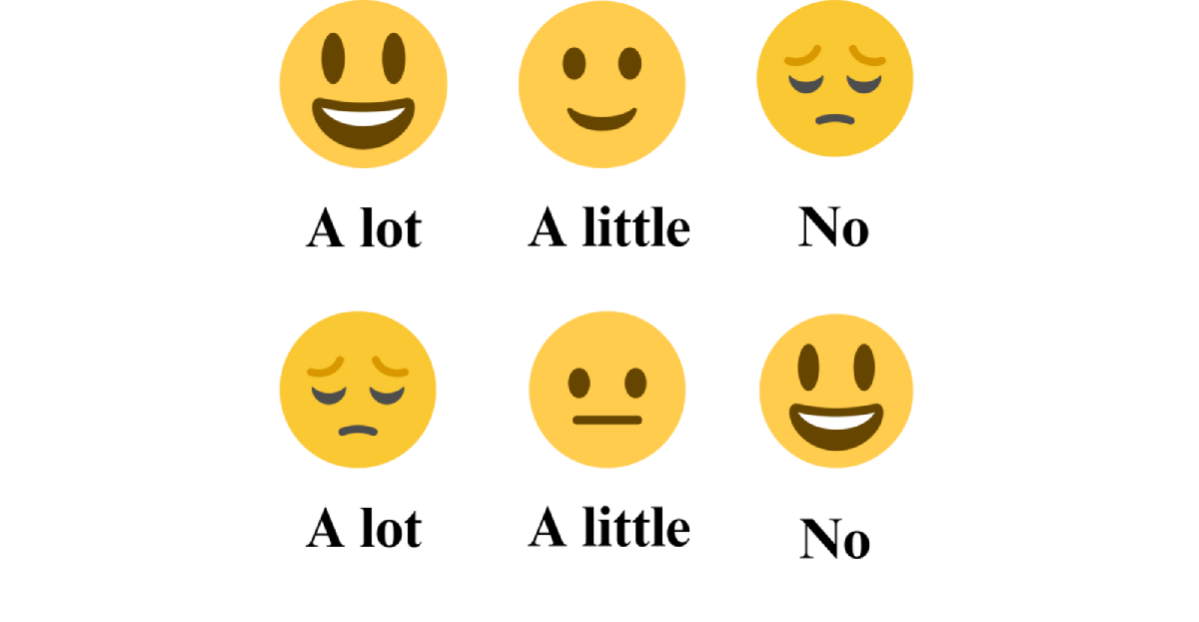
Emoticon card used as a visual aid to simplifying Likert-type assessment scales in a pilot study involving 8 adolescents with mild intellectual disabilities (October 2023 to May 2024). Each of the 3 response options was paired with a corresponding emoticon to enhance comprehension, with a modified set of emoticons used for the reversely structured question number 5.

Self-reported answers on the gameplay experience questionnaire [24] were assessed using a 3-point Likert scale. Respondents were asked to indicate the extent to which they agreed with each statement by selecting one of the following options: “A Lot,” “A Little,” or “No.” Because adults and children with ID often struggle with text comprehension, simplifying assessment scales is a frequent practice [25-27]. To improve understanding of the Likert-type items in this study, 2 strategies were used: each of the 3 response options was paired with a corresponding emoticon, and the researcher also presented the questions orally. The emoticon card is shown in Figure 2. For question number 5, different emoticons were used due to the reverse structure of the question. To assess usability in the context of VR, the focus should be on areas such as ease to use, feeling safe, and cumbersome handling [28,29].

The gameplay experience questionnaire included the following seven items: question 1, “Did you get used to the game quickly?”; question 2, “Were the controls easy to use?”; question 3, “Did the things you saw look real?”; question 4, “Were the VR goggles comfortable?”; question 5, “Were you worried about putting on the VR goggles?”; question 6, “Did it feel like you were in control?”; and question 7, “Did the way you moved look real?”

The gameplay experience questionnaire included seven items: getting used to the game (question 1), ease of controls (question 2), visual realism (question 3), comfort of VR goggles (question 4), concerns of VR goggles (question 5), sense of control (question 6) and realism of movements (question 7). To derive an overall gameplay experience score, the numerical ratings (1=”No,” 2=”A Little,” and 3=”A Lot”) from the 7 items were summed, with the score for question number 5 (“Were you worried about putting on the VR goggles?”) being reverse-coded prior to summation (ie, “A Lot” mapped to 1, “A Little” to 2, and “No” to 3), ensuring that a higher total score consistently reflected a more positive overall experience.

### Statistical Analysis

Calculations were performed using Statistica 13.0 software (TIBCO Software Inc). To assess whether the WISH and WON protocols induced statistically significant improvements: Wilcoxon signed-rank tests were used to analyze changes in independence levels (from the No problem! questionnaire) and exergame performance (number of stars earned); McNemar tests were used for analyzing changes in categorical data from the gameplay experience questionnaire; chi-square tests were used for other categorical comparisons, where appropriate; and Spearman rank-order correlations were performed to investigate relationships between participants’ independence levels (data from the No problem! questionnaire) and their exergame performance (number of stars earned) and overall gameplay experience (the final composite score from the gameplay experience questionnaire).

## Results

### WISH

Understanding the task in” W” step was assessed by the trainer after the warm-up. The data for each session in WISH protocol are presented in Table 1. During session 1, the results were as follows: median 1.0 (IQR 0.25; 0.75–1.00) point. This means that there were participants (6/8, 75%) who partially understood the instructions (and therefore received 1.00 point), but there were also participants (2/8, 25%) who did not understand the instructions at all (0.00 point). The score increased from session 1 to session 4. In session 4, all participants scored 2.00 points (8/8, 100%). A Wilcoxon signed-rank test was performed to compare the results of the participants in the WISH protocol between session 1 and session 4. The *P* value was .009 and the effect size was 0.87 for warm-up instruction understanding.

Understanding the task in “I” step was assessed by the trainer after completing the session in front of the projector. The data for each session in the WISH protocol are presented in Table 2. During session 1, the results were as follows: median 0.50 (IQR 1.00; 0.00–1.00), range (minimum-maximum) 0.00-1.00 point. This means that there were participants (4/8, 50%) who partially understood the instructions (and therefore received 1.00 point), but there were also participants (4/8, 50%) who did not understand the instructions at all (0.00 point). The mean score increased from session 1 to session 4. In session 4, all participants scored 2.00 points.

The star achievement in the “H” step was assessed by the trainer after VR exergame gameplay. The data for each session in the WISH protocol are presented in Table 3. During session 1, the results were as follows: median 0.75 (IQR 0.50; 0.50–1.00) points. This means that there were participants (4/8, 50%) who scored 1 full star (1.00 point), but there were also participants (4/8, 50%) who scored only half a star (0.50 point). The stars provided information about the result achieved in the VR exergame gameplay—the higher the score, the more stars summarizing the gameplay experience. The median score increased from session 1 to session 4. In session 4, the results were as follows:median 1.00 (IQR 0.25; 1.00–1.25) points, range (minimum-maximum) 0.50-2.00 points. One participant scored 2 points, one participant scored 1.5 points, 5 participants scored 1 point, and only one participant who completed the protocol scored 0.5 points.

**Table 1:** Trainer’s assessment of participants’ understanding of warm-up instructions during the “W” (Warm-up) step of the WISH (Warm-up, Imitation, Settings, Half-hour exergame session) protocol across sessions 1-4 in a pilot study involving adolescents with mild intellectual disabilities (N=8). Data collected from a special school in Poland (October 2023 to May 2024) are presented as median (IQR; Q1–Q3), mean (SD), and range for each session. The Wilcoxon signed-rank test performed to compare session 1 and session 4 revealed a statistically significant difference (*P*=.009) with a corresponding large effect size (r=0.87).

Variable			Session 1		Session 2		Session 3		Session 4
**Understanding of warm-up instructions, points^a^**									
	Median (IQR; Q1–Q3)	1.00 (0.25; 0.75–1.00)		1.00 (0.00; 1.00–1.00)		2.00 (0.25; 1.75–2.00)		2.00 (0.00; 2.00–2.00)	
	Mean (SD)	0.75 (0.46)		1.13 (0.35)		1.75 (0.46)		2.00 (0.00)	
	Range	0.00-1.00		1.00-2.00		1.00-2.00		2.00-2.00	
**Number of participants achieving score, n (%)**									
	0.00 points (No)	2 (25)		0 (0)		0 (0)		0 (0)	
	1.00 points (Partially)	6 (75)		7 (87)		2 (25)		0 (0)	
	2.00 points (Yes)	0 (0)		1 (13)		6 (75)		8 (100)	

^a^It indicates that the trainer recorded on the task sheet whether each participant understood the instructions and performed the warm-up correctly (yes=2 points, partially=1 point, and no=0 points).

**Table 2:** Trainer’s assessment of participants’ understanding of imitation instructions during the “I” (Imitation) step of the WISH (Warm-up, Imitation, Settings, Half-hour exergame session) protocol across sessions 1-4 in a pilot study involving adolescents with mild intellectual disabilities (N=8). Data collected from a special school in Poland (October 2023 to May 2024) are presented as median (IQR; Q1–Q3), mean (SD), and range for each session. The Wilcoxon signed-rank test performed to compare session 1 and session 4 revealed a statistically significant difference *P*=.007 with a corresponding large effect size (r=0.91).

Variable		Session 1	Session 2	Session 3	Session 4
**Understanding of imitation instructions, points^a^**					
	Median (IQR; Q1–Q3)	0.50 (1.00; 0.00–1.00)	1.00 (0.50; 1.00–1.50)	2.00 (0.00; 2.00–2.00)	2.00 (0.00; 2.00–2.00)
	Mean (SD)	0.50 (0.53)	1.25 (0.46)	1.86 (0.35)	2.00 (0.00)
	Range	0.00-1.00	1.00-2.00	1.00-2.00	2.00-2.00
**Number of participants achieving score, n (%)**					
	0.00 points (No)	4 (50)	0 (0)	0 (0)	0 (0)
	1.00 points (Partially)	4 (50)	6 (75)	1 (13)	0 (0)
	2.00 points (Yes)	0 (0)	2 (25)	7 (87)	8 (100)

^a^It indicates that the trainer recorded on the task sheet whether each participant understood the instructions and performed the warm-up correctly (yes=2 points, partially=1 point, and no=0 points).

**Table 3:** Trainer’s assessment of participants’ exergame performance (star achievement) during the “H” (Half-hour exergame session) step of the WISH (Warm-up, Imitation, Settings, Half-hour exergame session) protocol across sessions 1-4 in a pilot study involving adolescents with mild intellectual disabilities (N=8). Data collected from a special school in Poland (October 2023 to May 2024) are presented as median (IQR; Q1–Q3), mean (SD), and range for each session. The Wilcoxon signed-rank test performed to compare session 1 and session 4 revealed a statistically significant difference (*P*=.03) with a corresponding large effect size (r=0.90).

Variable		Session 1	Session 2	Session 3	Session 4
**Exergame performance (stars), points^a^**					
	Median (IQR; Q1–Q3)	0.75 (0.50; 0.50–1.00)	1.00 (0.50; 0.75–1.25)	1.00 (0.25; 0.75–1.00)	1.00 (0.25; 1.00–1.25)
	Mean (SD)	0.75 (0.27)	1.06 (0.52)	1.00 (0.53)	1.13 (0.44)
	Range	0.50-1.00	0.50-2.00	0.50-2.00	0.50-2.00
**Number of participants achieving score, n (%)**					
	0.00 points	0 (0)	0 (0)	0 (0)	0 (0)
	0.50 points	4 (50)	2 (25)	2 (25)	1 (13)
	1.00 points	4 (50)	4 (50)	5 (63)	5 (63)
	1.50 points	0 (0)	1 (13)	0 (0)	1 (13)
	2.00 points	0 (0)	1 (13)	1 (13)	1 (13)

^a^It indicates that the stars achievement system ranged from 0 to 5 (including half points), reflecting the result achieved in the VR exergame gameplay (higher scores indicate more stars and better performance).

### WON

Understanding the task in the “W” step was assessed by the trainer after the warm-up. The data are presented in Table 4. During session 5, the results were as follows: median 2.00 (IQR 1.00; 1.00–2.00) points, range (minimum-maximum) 1.00-2.00 points. This means that there were still participants (3/8, 37.50%) who partially understood the instructions (and therefore received 1.00 point), but there were no participants (n=0) who did not understand the instructions at all. The median score increased from session 5 to session 9. There were no changes from session 9 to session 16. In session 9, all participants scored 2.00 points (8/8, 100%).

The star achievement in the “O” step was assessed by the trainer after the VR exergame gameplay. The data for each session in WON protocol are presented in Table 5. During session 5, the results were as follows: median 1.00 (IQR 0.50; 1.00–1.50) points, range (minimum-maximum) 0.50-2.00 points. This means that there were participants (2/8, 25%) who scored 2 full stars (2.00 points), but there was also one participant who scored only half a star (0.50 points). The stars provided information about the result achieved in the VR exergame gameplay (the higher the score, the more stars that summarized the gameplay experience). The mean score increased from session 5 to session 16. In session 16, the results were as follows: median 4.00 (IQR 0.75; 3.75–4.50) points, range (minimum-maximum) 3.50-5.00 points. This means that there was one participant who scored 5 full stars (5.00 points), which was the maximum that could be obtained. Two participants received 4.5 points, 3 participants received 4 stars, and 2 participants received 3.5 stars. This was the score with which they concluded the study.

The gameplay experience questionnaire shows the fluctuation of participants’ VR exergame gameplay experience between the fourth and last sessions. The questionnaire was divided into 7 sections: getting used to the game (question 1), ease of controls (question 2), visual realism (question 3), comfort of VR goggles (question 4), concerns of VR goggles (question 5), sense of control (question 6), and realism of movements (question 7). The data for each item by session are presented in Table 6.

A Wilcoxon signed-rank test was performed to compare participants’ scores on understanding instructions within the WON protocol between session 5 and session 16. The analysis revealed that the observed improvement was statistically significant (*P*=.01), with a very large effect size (*r*=0.87). This confirms that the protocols effectively enhanced participants’ independence and ability to navigate the virtual environment. To assess the influence of the protocols on gameplay experience, a McNemar test was performed on questionnaire data, comparing responses from session 5 and session 16. The analysis showed a statistically significant improvement in several aspects of perceived game usability. Participants adapted to the game more quickly (*P*=.01), found the controls easier to use (*P*=.01), and felt more in control of their virtual avatar (*P*=.01). Additionally, there was a significant reduction in the level of worry associated with using VR goggles (*P*=.02) and a significant improvement in their perceived comfort (*P*=.01). Conversely, changes in perceived visual realism (*P*=.16) and movement realism (*P*=.32) were not statistically significant. These findings provide crucial quantitative evidence supporting the positive impact of the protocols on user experience.

The No problem! questionnaire was an independence gameplay questionnaire combined with interviews and included 7 functional levels of independence, ranging from total assistance to total independence. Data on independence in gameplay level were collected from sessions 5 to 16 (the WON protocol, “N” step) and are presented in Table 7.

A Wilcoxon signed-rank test was conducted to assess the impact of the WON protocol on participants’ level of independence in gameplay by comparing data from session 5 and session 16. The analysis revealed that the observed improvement in independence levels was statistically significant (*P*=.008). This value is well below the standard .05 significance threshold, confirming that the increase in independence was not due to random chance. Furthermore, the effect size (*r*=0.89) indicates a very large effect of the intervention, providing strong quantitative evidence of its significant impact on developing participants’ autonomy.

To investigate the relationship between the improvement in participant independence and their gameplay experience, a Spearman rank-order correlation analysis was conducted. The analysis compared the final scores from the No problem! independence questionnaire with the final scores from the gameplay experience questionnaire. The results revealed a very strong and statistically significant correlation between the two variables.

As shown in Table 8, the high Spearman rank-order correlation coefficients for most items indicate that as participants’ independence increased, so did their positive assessment of the VR exergame. This provides strong evidence that the protocol not only enhanced autonomy but also significantly improved the overall user experience. Furthermore, a Spearman rank-order correlation between the participants’ independence levels and the aggregated overall gameplay experience score revealed a moderately strong and statistically significant positive association (ρ=0.63; *P*<.05).

A Spearman rank-order correlation was conducted to investigate the relationship between participants’ independence levels (data from the No problem! questionnaire) and their exergame performance (number of stars earned). This analysis used data from the final session (session 16) for all 8 participants. The analysis revealed a statistically significant positive correlation (*P*=.002). The high correlation coefficient (ρ=0.91) indicates that as participants became more independent during the exergaming session, their exergame achievements also consistently improved.

**Table 4:** Trainer’s assessment of participants’ understanding of warm-up instructions during the “W” (Warm-up) step of the WON (Warm-up, Objective evaluation, No problem!) protocol across sessions 5-16 in a pilot study involving adolescents with mild intellectual disabilities (N=8). Data collected from a special school in Poland (October 2023 to May 2024) are presented as median (IQR; Q1–Q3), mean (SD), and range for each session, along with the distribution of participants achieving specific points. The Wilcoxon signed-rank test performed to compare session 5 and session 16 revealed a statistically significant difference (*P*=.06) with a corresponding large effect size (r=0.87).

Variable		Session 5	Sessions 9-16
**Understanding of warm-up instructions, points^a^**			
	Median (IQR; Q1–Q3)	2.00 (1.00; 1.00–2.00)	2.00 (0.00; 2.00–2.00)
	Mean (SD)	1.63 (0.48)	2.00 (0.00)
	Range (minimum-maximum)	1.00-2.00	2.00-2.00
**Number of participants achieving points, n (%)**			
	0.00 points (No)	0 (0)	0 (0)
	1.00 points (Partially)	3 (37.5)	0 (0)
	2.00 points (Yes)	5 (62.5)	8 (100)

^a^It indicates that the trainer recorded on the task sheet whether each participant understood the instructions and performed the warm-up correctly (yes=2 points, partially=1 point, and no= 0 points).

**Table 5:** Trainer’s assessment of participants’ exergame performance during the “O” (Objective evaluation) step of the WON (Warm-up, Objective evaluation, No problem!) protocol across sessions 5-16 in a pilot study involving adolescents with mild intellectual disabilities (N=8). Data collected from a special school in Poland (October 2023 to May 2024) are presented as median (IQR; Q1–Q3), mean (SD), and range for each session, along with the distribution of participants achieving specific points. The Wilcoxon signed-rank test performed to compare session 5 and session 16 revealed a statistically significant difference (*P*=.008) with a corresponding large effect size (r=0.89).

Variable		Session 5	Session 16
**Exergame performance (stars), points^a^**			
	Median (IQR; Q1–Q3)	1.00 (0.50; 1.00–1.50)	4.00 (0.75; 3.75–4.50)
	Mean (SD)	1.19 (0.53)	4.13 (0.52)
	Range	0.50-2.00	3.50-5.00
**Number of participants achieving points, n (%)**			
	0.50 points	1 (13)	0 (0)
	1.00 points	5 (62)	0 (0)
	1.50 points	0 (0)	0 (0)
	2.00 points	2 (25)	0 (0)
	2.50 points	0 (0)	0 (0)
	3.00 points	0 (0)	0 (0)
	3.50 points	0 (0)	2 (25)
	4.00 points	0 (0)	3 (38)
	4.50 points	0 (0)	2 (25)
	5.00 points	0 (0)	1 (13)

^a^It indicates that the stars achievement system ranged from 0 to 5 (including half points), reflecting the result achieved in the VR exergame gameplay (higher scores indicate more stars and better performance).

**Table 6:** Participants’ self-reported gameplay experience during the “N” (No problem! questionnaires) step of the WON (Warm-up, Objective evaluation, No problem!) protocol, comparing session 5 and session 16, in a pilot study involving adolescents with mild intellectual disabilities (N=8). Data collected from a special school in Poland (October 2023 to May 2024) are presented as n (%). Results of the Wilcoxon signed-rank test comparing session 5 and session 16 are also shown.

Questions and responses		Session 5, n (%)	Session 16, n (%)	Comparison (session 5 vs session 16), *P* value	
**Question 1: Did you get used to the game quickly?**					.01^a^
	A lot	0 (0)	5 (62.5)		
	A little	2 (25)	3 (37.5)		
	No	6 (75)	0 (0)		
**Question 2: Were the controls easy to use?**					.01^a^
	A lot	0 (0)	6 (75)		
	A little	2 (25)	2 (25)		
	No	6 (75)	0 (0)		
**Question 3: Did the things you saw look real?**					.15^b^
	A lot	1 (12.5)	3 (37.5)		
	A little	5 (62.5)	5 (62.5)		
	No	2 (25)	0 (0)		
**Question 4: Was the VR^c^** **goggles comfortable?**					01^a^
	A lot	1 (12.5)	7 (87.5)	.	
	A little	1 (12.5)	1 (12.5)		
	No	6 (75)	0 (0)		
**Question 5: Were you worried about putting on the VR goggles?**					.02^a^
	A lot	6 (75)	0 (0)		
	A little	1 (12.5)	2 (25)		
	No	1 (12.5)	6 (75)		
**Question 6: Did it feel like you were in control?**					.01^a^
	A lot	0 (0)	7 (87.5)		
	A little	2 (25)	1 (12.5)		
	No	6 (75)	0 (0)		
**Question 7: Did the way you moved look real?**					.32^b^
	A lot	2 (25)	7 (87.5)		
	A little	5 (62.5)	1 (12.5)		
	No	1 (12.5)	0 (0)		

^a^Statistically significant at *P*<.05.

^b^Not statistically significant at *P*<.05.

^c^VR: virtual reality.

**Table 7:** Trainer’s assessment of participants’ independence in gameplay during the “N” (NO problem! questionnaires) step of the WON (Warm-up, Objective evaluation, No problem!) protocol across sessions 5-16 in a pilot study involving adolescents with mild intellectual disabilities (N=8). Data collected from a special school in Poland (October 2023 to May 2024) are presented as median (IQR; Q1–Q3), mean (SD), and range for each session. The Wilcoxon signed-rank test performed to compare session 5 and session 16 revealed a statistically significant difference (*P*=.008) with a corresponding large effect size (r=0.89).

Variable		Session 5	Session 16
**Independence in gameplay level, points^a^**			
	Median (IQR; Q1–Q3)	4.00 (0.00)	7.00 (1.00)
	Mean (SD)	4.13 (0.35)	6.63 (0.52)
	Range	4.00-5.00	6.00-7.00
**Number of participants achieving points, n (%)**			
	1.00 points (total assistance)	0 (0)	0 (0)
	2.00 points (maximum assistance)	0 (0)	0 (0)
	3.00 points (moderate assistance)	0 (0)	0 (0)
	4.00 points (minimal assistance)	7 (87.5)	0 (0)
	5.00 points (supervision)	1 (12.5)	0 (0)
	6.00 points (incomplete independence)	0 (0)	7 (87.5)
	7.00 points (total independence)	0 (0)	1 (12.5)

^a^It refers to a 7-point scale (1-7) defined as follows: 7 points=total independence, 6 points=incomplete independence, 5 points=supervision, 4 points=minimal assistance, 3 points=moderate assistance, 2 points=maximum assistance, and 1 point=total assistance.

**Table 8:** Spearman rank-order correlation coefficients examining the relationship between participants’ final independence levels and their final gameplay experience, in a pilot study involving adolescents with mild intellectual disabilities (N=8). Data collected from a special school in Poland (October 2023-May 2024) are shown. Statistical significance was assessed using the Spearman rank-order correlation test.

Gameplay experience questionnaire item	Spearman correlation coefficient, ρ	*P* value
Question 1: Did you get used to the game quickly?	0.94	.001^a^
Question 2: Were the controls easy to use?	0.91	.002^a^
Question 3: Did the things you saw look real?	0.69	.058^b^
Question 4: Were the VR goggles comfortable?	0.97	.001^a^
Question 5: Were you worried about putting on the VR goggles?	0.86	.006^a^
Question 6: Did it feel like you were in control?	0.94	.001^a^
Question 7: Did the way you moved look real?	0.85	.008^a^

^a^Statistically significant at *P*<.05.

^b^Not statistically significant at *P*<.05.

## Discussion

### Principal Results

This pilot study aimed to evaluate the effectiveness of the WISH and WON training protocols in enhancing the independence, performance, and overall gameplay experience of individuals with mild ID using a VR exergame. The findings suggest that a structured training methodology may be highly beneficial for this population. The results from the WISH protocol indicate its success in establishing foundational skills. The statistically significant improvements in participants’ understanding of warm-up and imitation instructions (*P*=.009 and *P*=.007, respectively) with very large effect sizes (*r*=0.87 and *r*=0.91) suggest that the initial 4 sessions helped acclimate participants to the game’s core mechanics and rules. This structured introduction appears to be a crucial prerequisite for successful, independent engagement. This is further supported by the significant increase in in-game exergame performance (*P*=.03). The protocol’s emphasis on developing correct movement technique and preventing unhealthy posture (eg, slouching to catch a coin) appeared to be critical to ensuring both safety and proper skill acquisition.

The WON protocol appeared to successfully build upon this foundation to foster independence. We observed a statistically significant increase in participants’ exergame performance from session 5 to session 16 (*P*=.008), with a large effect size (*r*=0.89). This finding suggests that the protocol’s gradual reduction of trainer assistance may have effectively facilitated a shift from supported engagement to autonomous performance. The correlation between increased independence and better exergame performance (ρ=0.91; *P*=.002) provides quantitative evidence that developing autonomy was associated with better performance outcomes. The clear progression in independence, from requiring minimal assistance in the initial sessions to achieving complete independence in the final sessions, underscores the protocol’s potential in promoting self-reliance. This is especially important as the ability to manage one’s own affairs and rely on personal effort and judgment is a key component of real-life independence.

The protocols also appeared to have a profound positive impact on the subjective user experience. A strong correlation was found between increased independence and a more positive overall gameplay experience (ρ=0.63; *P*<.05). This may indicate that the protocols not only improved objective skills but also built confidence and comfort in the virtual environment. This is consistent with previous research suggesting that adapting gameplay to individual capabilities is essential for a positive experience [11,30-32]. Our findings suggest that the OhShape! VR exergame, with its simple controls and full-body movement requirements, combined with the structured protocols, was well-suited for this population, potentially leading to significant improvements in perceived comfort, ease of controls, and a sense of control over the virtual avatar.

These findings have important implications for inclusive PE. The structured WISH and WON protocols may offer an evidence-based framework for integrating VR exergames into PE lessons for individuals with mild ID. By prioritizing procedural learning and independence, these protocols could help this population overcome a key barrier to PA.

### Comparison With Previous Work

While a growing body of literature supports the use of VR exergames to promote PA and motor skills among individuals with ID, there remains a significant gap in research concerning structured, pedagogical methodologies for technology adoption. This study contributes to the field by not only reinforcing the potential benefits of VR exergaming but, more importantly, by proposing a concrete, evidence-based training framework for achieving independence.

Our findings appear consistent with previous work demonstrating that VR exergames are a highly engaging and motivating tool for individuals with ID, capable of increasing PA participation [33-35]. However, a key novelty of our research lies in the systematic approach taken. Most existing studies have focused on the outcomes of VR use, such as changes in physical fitness or energy expenditure, without detailing the structured process of teaching the technology itself. The WISH and WON protocols seek to explore ways to address this gap by offering a replicable, step-by-step methodology that aims to tackle the foundational challenges of task comprehension and motor learning in this population.

The systematic naming of our training methodologies as the WISH and WON protocols was a deliberate choice intended to enhance their clarity, memorability, and potential for widespread adoption. The use of acronyms as a tool for structuring complex information is a well-established practice across scientific disciplines, particularly in medicine and education, where it facilitates the memorization and application of multistep procedures. For example, the patient handover protocol described by Hanke et al [36] uses the ISBAR (Introduction, Situation, Background, Assessment, and Actions) acronym to ensure a structured and comprehensive communication process. Similarly, the names of our protocols serve as easy-to-recall frameworks for teachers or therapists, summarizing the core steps of each training phase. Given that the target population—individuals with mild ID—benefits significantly from clear, concise, and repeatable instructions, the acronyms WISH and WON provide a simple and effective mnemonic device.

In summary, while the therapeutic potential of VR exergaming for individuals with ID is well documented [37], this pilot study aims to provide a unique and essential contribution by offering a transferable scientific pattern. The WISH and WON protocols appear to serve as a practical teaching model that could be applied to various VR exergames, addressing the critical need for structured educational frameworks in this domain. Our findings may highlight that beyond simply providing access to technology, a well-designed pedagogical approach could be essential for cultivating true independence and maximizing the benefits of VR-based interventions.

### Limitations

It is important to acknowledge the limitations of this study. First, the small sample size (N=8) inherently limits the generalizability of our findings. This small sample may also have influenced the statistical power, potentially affecting the significance of some results and making them not fully representative of the wider population of individuals with mild ID. As a pilot study, our primary aim was to test the feasibility and initial effectiveness of our protocols in a targeted group; thus, a larger and more representative sample was beyond the scope of this initial investigation. To address this in future research, replication with substantially larger sample sizes will be crucial, potentially through multisite collaborations, to increase generalizability and statistical robustness.

Second, the absence of a control group in this design is a significant limitation. This design prevents us from definitively isolating the effects of the WISH and WON protocols from other factors, such as natural learning or repeated practice. Furthermore, because participants were aware of their involvement in a novel intervention, the Hawthorne effect [38,39]—in which individuals modify their behavior in response to being observed—cannot be entirely ruled out as a contributing factor to the observed improvements. Consequently, while promising improvements were observed, we cannot conclusively state that they were solely caused by our intervention. This limitation was deemed acceptable for this pilot study, in which the primary focus was on assessing the feasibility and initial efficacy of the protocols in a small, targeted group rather than on direct comparison with traditional training methods. However, it is worth noting that the intervention sessions were integrated into the standard PE curriculum, occurring approximately once every 2 weeks from October to May and interspersed with regular PE lessons. This intermittent schedule, rather than continuous daily exposure, may have partially mitigated the extent of natural learning or practice effects that might occur with more frequent, concentrated interventions. Despite these partial mitigations, our findings should be interpreted with caution regarding causality. To improve upon this, future research should implement randomized controlled trial designs to provide more robust evidence of the protocols’ direct impact and to establish stronger causal links.

Third, the study’s evaluation was conducted at a single institution—a special school complex in Poland. This geographic and institutional specificity limits the external validity of the findings, as the unique characteristics of this setting (eg, educational philosophy, available resources, specific student demographics, and cultural factors) may not be fully replicable elsewhere. To mitigate this in future research, the protocols should be replicated in diverse populations and settings, including different countries, school types, and therapeutic environments, to assess their generalizability across various contexts.

Fourth, the wide age range of participants (15-20 years) may introduce significant developmental variability within this population with ID. This age range was chosen partly on the premise that the most rapid phases of morphofunctional maturation [40] are generally less pronounced in late adolescence compared with younger childhood, which might provide a more stable baseline for evaluating the acquisition of new motor skills and independence through VR interventions. Nevertheless, this age variability still acts as a potential confounding factor that was not specifically controlled for or discussed in detail within this pilot study. While acknowledging this as a limitation, the homogeneity of the participants’ mild intellectual disability level provided a baseline for consistent instruction. Future research should investigate narrower age cohorts or include age as a covariate in the analysis to better understand its potential influence on outcomes and tailor interventions accordingly.

Fifth, while the assessment of understanding instructions during the WISH protocol used a structured task sheet with predefined, detailed criteria for a 3-point rating scale, the data were collected by a single trainer. This reliance on a sole observer, despite the established rubric, introduces a potential for observer bias and limits the ability to assess interrater reliability. Consequently, the full objectivity of these specific findings cannot be fully ascertained. To enhance methodological rigor in future studies, it is crucial to incorporate multiple independent raters with demonstrated interrater agreement, using a comprehensive training protocol for raters to improve the objectivity and reliability of such observational data.

Sixth, the No problem! questionnaire used an adapted 7-level functional independence scale derived from the FIM levels. Although the FIM is an established tool, the specific adaptation of its levels and their application within our No problem! questionnaire for assessing exergame independence were not been subjected to separate validation or reliability measurements within the context of this study. Future research using this adapted methodology should conduct thorough psychometric evaluations to ensure its validity and reliability for the specific population and research context.

Finally, the novelty of VR exergaming in the study setting constitutes another limitation. In Poland, VR goggles are not yet widely used, and many participants had no previous experience with VR. Although this novelty likely contributed to high initial engagement, it also suggests that some observed improvements could be partly attributed to mere exposure to new technology rather than solely to the structured protocols. This limitation was partially mitigated by the WISH protocol’s specific focus on familiarizing participants with the VR environment and the game’s core mechanics. Future studies conducted in settings where VR technology is more commonplace, or those using a longer familiarization phase, could help to more clearly differentiate the effects of novelty from those of the intervention itself.

Despite these limitations, the findings from this pilot study may offer initial insights into the feasibility, acceptability, and potential benefits of the WISH and WON protocols. They may help inform the design of future, more robust, and controlled studies by highlighting areas for improvement and further investigation regarding participant engagement and performance within a VR exergame context for individuals with mild ID.

### Implications and Future Work

In summary, this pilot study provides a unique and essential contribution by suggesting a transferable scientific pattern. The WISH and WON protocols appear to serve as a practical teaching model that could be applied to a variety of VR exergames, addressing the critical need for structured educational frameworks in this domain. Our findings highlight that, beyond simply providing access to technology, a well-designed pedagogical approach may be essential for cultivating true independence and maximizing the benefits of VR-based interventions.

For physical education teachers and therapists working with individuals with mild ID, our findings suggest a structured, phased approach to introducing VR exergames. These recommendations align with widely accepted principles of motor learning and skill acquisition [41-43]. This approach is particularly relevant given that children and adolescents with ID often exhibit slower motor learning and require more time and repetitions to acquire new tasks compared with typically developing peers [43]. Based on the WISH and WON protocols, key recommendations include foundational skill building (by WISH): beginning with a dedicated phase focused on familiarization with the VR environment, basic game mechanics, and explicit instruction on fundamental movements (eg, understanding warm-up cues, precise imitation of actions, maintaining correct posture). Crucially, warm-up exercises should incorporate movements and terminology directly related to the VR exergame, ensuring that participants are familiar with the required actions and verbal cues before donning the VR goggles for the main session. This initial phase, embodying the principle of teaching from easier to more difficult tasks, should prioritize task comprehension and motor learning over performance. Gradual independence fostering (by WON): systematically reducing assistance as participants demonstrate mastery, progressing from full support to verbal cues and then to independent play, encouraging self-reliance and autonomous problem-solving within the game. This approach reflects the progressive nature of motor skill development and the fading of external support. Emphasis on procedural learning (by WISH and WON): recognizing that individuals with mild ID often benefit from clear, repetitive, and consistent instructions and that complex movements should be broken down into smaller, manageable steps. Integration of feedback and reinforcement (by WISH and WON): providing constructive feedback alongside positive reinforcement. Monitoring beyond performance (by WISH and WON): paying attention not only to in-game scores but also to indicators of independence, comfort with the VR technology, and overall enjoyment of the experience.

However, it is crucial to acknowledge the potential barriers to the widespread implementation of VR exergaming in educational or therapeutic settings. These include the significant initial cost of VR hardware and software, issues of accessibility for institutions with limited budgets or infrastructure, and the need for adequate technological literacy among both educators or therapists and participants. Addressing these practical challenges will be vital for the successful translation of these protocols into broader practice.

While this pilot study observed improvements in gameplay independence, the direct transferability of these skills to real-world daily tasks remains a hypothesis for future research. We speculate that by applying the WISH and WON protocols, teachers or therapists might be able to guide individuals with mild ID to achieve gameplay independence across various VR exergames involving similar full- or partial-body movements. This approach could potentially foster greater engagement in PA and may facilitate the translation of learned movements (eg, correct squat, no slouching) into practical daily life, thereby cultivating greater overall independence. Further studies are indeed needed to fully understand the long-term benefits of VR-based exercise on physical fitness and functional independence in individuals with ID.

### Conclusions

This pilot study suggests that the structured WISH and WON training protocols may have the potential to enhance functional autonomy, exergame performance, and overall gameplay experience in individuals with mild ID.

## Appendix

Multimedia Appendix 1 Video of step “I” (Imitation) in the WISH (Warm-up, Imitation, Settings, Half-hour exergame session) protocol.

Multimedia Appendix 2 Functional Independence Measure (FIM) questionnaire (original version).

## Abbreviations

FIM: Functional Independence Measure
ID: intellectual disabilities
IQ: intelligence quotient
ISBAR: Introduction, Situation, Background, Assessment, and Actions
PA: physical activity
PE: physical education
VR: virtual reality
WISH: Warm-up, Imitation, Settings, Half-hour exergame session
WON: Warm-up, Objective evaluation, No problem!

## Data Availability

The datasets generated or analyzed during this study are available from the corresponding author on reasonable request.
