# Supplementary material for: Achieving Gameplay Independence in Virtual Reality Exergames for Individuals With Mild Intellectual Disabilities: Pilot Study
Source: JMIR Serious Games. 2025 Nov 14;13:e71823. doi: 10.2196/71823 (PMC12663707; doi:10.2196/71823)
Supplement: Multimedia Appendix 2 [file games_v13i1e71823_app2.pdf]

FIM™ instrument

LEVELS		
	7 Complete Independence (Timely, Safely)<br>6 Modified Independence (Device)	**NO HELPER**
	**Modified Dependence**<br>5 Supervision (Subject = 100%+)<br>4 Minimal Assist (Subject = 75%+)<br>3 Moderate Assist (Subject = 50%+)<br><br>**Complete Dependence**<br>2 Maximal Assist (Subject =25%+)<br>1 Total Assist (Subject = less than 25%)	**HELPER**

	ADMISSION	DISCHARGE	FOLLOW-UP
**Self-Care**			
A. Eating			
B Grooming			
C. Bathing			
D. Dressing - Upper Body			
E. Dressing - Lower Body			
F. Toileting			
**Sphincter Control**			
G. Bladder Management			
H. Bowel Management			
**Transfers**			
I. Bed, Chair, Wheelchair			
J. Toilet			
K. Tub, Shower			
**Locomotion**			
L. Walk/Wheelchair	W Walk / C Wheelchair / B Both	W Walk / C Wheelchair / B Both	W Walk / C Wheelchair / B Both
M. Stairs			
Motor Subtotal Score			
**Communication**			
N. Comprehension	A Auditory / V Visual / B Both	A Auditory / V Visual / B Both	A Auditory / V Visual / B Both
O. Expression	V Vocal / N Nonvocal / B Both	V Vocal / N Nonvocal / B Both	V Vocal / N Nonvocal / B Both
**Social Cognition**			
P. Social Interaction			
Q. Problem Solving			
R. Memory			
Cognitive Subtotal Score			
TOTAL FIM Score			

NOTE: Leave no blanks. Enter 1 if patient not testable due to risk

FIM™ Instrument. Copyright ©1997 Uniform Data System for Medical Rehabilitation, a divison of U B Foundation Activities, Inc.
Reprinted with the permission of UDSMR, University at Buffalo, 232 Parker Hall, 3435 Main Street, Buffalo, NY 14214.
